# Estrogen hormone is an essential sex factor inhibiting inflammation and immune response in COVID-19

**DOI:** 10.21203/rs.3.rs-936900/v1

**Published:** 2021-09-30

**Authors:** Fuhai Li, Adrianus C.M. Boon, Andrew P. Michelson, Randi E. Foraker, Ming Zhan, Philip R.O. Payne

**Affiliations:** 1 Institute for Informatics (I2), Washington University in St. Louis School of Medicine, St. Louis, MO, USA; 2 Department of Pediatrics, Washington University in St. Louis School of Medicine, St. Louis, MO, USA; 3 Department of Medicine, Washington University in St. Louis School of Medicine, St. Louis, MO, USA; 4 Pathology & Immunology, Washington University in St. Louis School of Medicine, St. Louis, MO, USA; 5 Department of Molecular Microbiology, Washington University in St. Louis School of Medicine, St. Louis, MO, USA; 6 Pulmonary and Critical Care Medicine, Washington University in St. Louis School of Medicine, St. Louis, MO, USA; 7 National Institute of Mental Health (NIMH), NIH, Bethesda, MD, USA.

**Keywords:** Estrogen hormone, sex factor, inflammation and immune response, COVID-19, vaccines

## Abstract

Although vaccines have been evaluated and approved for SARS-CoV-2 infection prevention, there remains a lack of effective treatments to reduce the mortality of COVID-19 patients already infected with SARS-CoV-2. The global data of COVID-19 showed that men have a higher mortality rate than women. We further observed that the proportion of mortality of female increases starting from around the age of 55 significantly. Thus, sex is an essential factor associated with COVID-19 mortality, and sex related genetic factors could be interesting mechanisms and targets for COVID-19 treatment. However, the associated sex factors and signaling pathways remain unclear. Here, we propose to uncover the potential sex associated factors using systematic and integrative network analysis. The unique results indicated that estrogen hormones (ER), e.g., estrone and estriol, 1) interacting with ESR1/2 receptors, 2) can inhibit SARS-CoV-2 caused inflammation and immune response signaling in host cells; and 3) estrogen hormone is associated with the distinct fatality rates between male and female COVID-19 patients. Specifically, a high level of estradiol protecting young female COVID-19 patients, and estrogen loss to an extremely low level in females after about 55 years of age causing the increased fatality rate of women. In conclusion, estrogen hormone, interacting with ESR1/2 receptors, is an essential sex factor that protects COVID-19 patients by inhibiting inflammation and immune response caused by SARS-CoV-2 infection. Medications perturb the down-stream of ESR1/ESR2 to inhibit the inflammation and immune response can be effective or synergistic combined with other existing drugs for COVID-19 treatment.

## Introduction

Globally, by September 25, 2021, over 230 million^1^ people were diagnosed with Coronavirus Disease 2019 (COVID-19), which is caused by the Severe Acute Respiratory Syndrome Coronavirus 2 (SARS-CoV-2). COVID-19 has a relatively high mortality rate^2^, and more than 4.7 million patients have died from the pandemic to-date^1^. The number of patients infected with SARS-CoV-2 and succumbing to COVID-19 and related complications are still increasing rapidly worldwide. Meanwhile, several vaccines have been approved for infection prevention, but may not prevent viral transmission to susceptible individuals. Further, to-date, Remdesivir remains the only FDA approved drug for COVID-19 treatment and it has shown variable efficacy. From the perspective of viral replication at the molecular level, Remdesivir is the most widely-used drug that specifically targets and inhibits the RNA-dependent RNA polymerase (RdRp), which is the critical target for viral replication. On May 1^st^, 2020, Remdesivir was granted an FDA Emergency Use Authorization (EUA) for COVID-19 treatment, and obtained full FDA approval on October 22, 2020. The final report from the most recent clinical trial of Remdesivir indicated that is can reduce the recovery time of COVID-19 patients to about 5 days, and that it reduces overall mortality rates. Though these results are promising, the effect of Remdesivir alone remains limited. In light of these challenges in terms of the prevention and treatment of COVID-19, many drugs and drug combinations are being tested on their own and in combination with Remdesivir in more than a thousand clinical trials globally. Among these drugs is dexamethasone, an FDA-approved drug, which was reported to be able to reduce the death rate of patients with severe COVID-19^3^. However, the death rate for such patients remains high despite such potential reductions.

Sex had been indicated as an essential factor in the mortality of COVID-19. The global data of COVID-19 showed that men have a higher mortality rate than women^4,5^. For example, the meta-analysis was conducted, and the analysis results showed that there were 3 times more male patients requiring intensive treatment unit admission than females, whereas there is a similar proportion of men and women with COVID-19 confirmation^5^. In addition, we further observed that the proportion of mortality of female increases starting from around the age of 55 significantly (see the results). Thus, the sex difference indicated that sex related genetic factors could be an interesting mechanism and targets for COVID-19 treatment, combined with existing treatment options. Numerous studies have been reported in the literature concerning efforts to understand the signaling mechanism and identify effective targets for COVID-19 from different perspectives^6,7,8,9,10,11,12,13^, like transcriptomic data analysis, proteomics data analysis, investigation of relevant protein structures, and the use of experimental and computational methodologies. However, few studies have been specifically designed for investigating sex related signaling pathways and targets. The underlying targets and signaling pathways associated with the sex difference remain unclear.

In response to the preceding gaps in knowledge, in this study, the objective is to uncover the potential sex associated factors using systematic and integrative network analysis. Specifically, we explore how protein-protein interaction screening data, transcriptomics data, signaling pathway data, protein-protein interaction signaling networks, and transcription factor-target regulatory networks can be integrated to enable such network analyses. We then go on to determine drug-target interactions and associations based upon an interrogate of the drugBank^14^ and connectivity map (CMAP) databases^15,16^ in order to identify the potentially-effective targets from the drug-target interaction perspective. Our unique results with novel discoveries, i.e., estrogen hormones (ER), like estrone and estriol, 1) interacting with ESR1/2 receptors, 2) can inhibit SARS-CoV-2 caused inflammation and immune response signaling in host cells; and 3) the loss of estrogen hormones to an extremely low level after 55 years of age is associated with the mortality increasing in female. These three novel observations support estrogen hormone is an essential sex factor causing the distinct fatality patterns between male and female COVID-19 patients.

## Materials and Methods

### Methodology Overview

**Figure 1:**
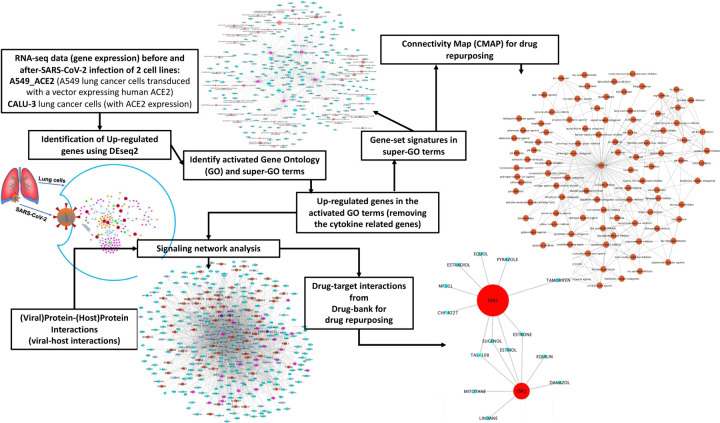
Overview of the proposed methodology.

Fig. 1 shows the overview of the proposed integrative data analysis model. Specifically, the raw counts of RNA-seq data (gene expression) of two lung cancer cell lines expressing ACE2 before and after SARS-CoV-2 infection were collected and used to identify commonly up-regulated genes using the DEseq2 model. Then gene ontology (GO) term enrichment analysis and super-GO term (consisting of a set of GO terms with similar functions) analysis were conducted to identify activated biological functions caused by SARS-CoV-2 infection using such up-regulated genes. Subsequently, up-regulated genes associated with super-GO terms were used as the gene set signatures in order to repurpose drugs using the connectivity map (CMAP) database, thus producing a list of top-ranked drugs that can potentially inhibit these up-regulated genes in the activated super GO terms. In parallel, the activated core signaling network caused by viral infection within host cells was constructed by integrating viral-host protein-protein interactions, the up-regulated genes associated with the activated GO terms, as well as the bioGrid protein-protein interaction network, and fold change values of all the genes as found in the RNA-seq data. Then, drug-target interaction information was derived from drugBank, and drugs that target the inferred signaling network proteins were identified as potential candidates for repurposing for SARS-CoV-2 replication inhibition and resultant COVID-19 treatment. The details of the related datasets and methods are described further in the following sections. All database and datasets used in this study are open and publicly available.

### RdRp protein complex and co-factors of SARS-CoV-2.

The 3D cryo-electron microscopy (cryo-EM) structure of the protein complex associated with RNA-dependent RNA polymerase (RdRp), i.e., nsp12, and its two co-factors, nsp7 and nsp8, of SARS-CoV-2, has been reported upon in the literature, including in the indicated references^17,18^. These structures are essential contributors of viral replication, working together with the transcription machinery of host cells. The RdRp is thought to be the primary target of the antiviral drugs, like Remdesivir.

### Proteins binding to nsp7, nsp8 and nsp12 (RdRp).

To identify the (prey) proteins binding to (bait) nsp7, nsp8 and nsp12 (and other viral proteins), protein-protein binding assays were conducted, as reported in reference^6^. Specifically, there are 20, 34, and 24 proteins interacting with nsp12, nsp7 and nsp8 respectively, and there is no overlap among these proteins. The data is available in reference^6^.

### Transcriptomic (gene expression) data analysis of two lung host cell lines, A549_ACE2 and CALU-3 cells, after SARS-CoV-2 infection.

The raw counts of RNA-seq data (transcriptomic response) of A549_ACE2 (engineered with expression of ACE2 protein), and CALU-3 lung cancer cells (with ACE2 expression) cells infected by SARS-CoV-2 were available in a single dataset (GSE147507) from the Gene Expression Omnibus (GEO) database^11^. The DEseq2^19^ approach was used to calculate the fold change and p-value of individual genes in the A549_ACE2, and CALU-3 lung cancer cells respectively before- and after-exposure to SARS-CoV-2 (with 3 replications). Then up-regulated genes were obtained by using fold change >=2.0 and p-value <= 0.05. Subsequently, the overlapping (intersection) genes between the up-regulated genes found in CALU-3 and A549_ACE2 cell lines were selected for further analysis as described below.

### Gene ontology (GO) enrichment analysis and super GO clustering analysis

The Fisher’s exact test was used to identify activated biological processes (BP) gene ontology (GO)^20^ terms associated with the identified up-regulated genes. Then, the activated GO terms with genes in [10, 500] and p-value <= 0.05 were identified. Among the hundreds of GO terms found, we further empirically selected the potentially virus-related GO terms. To further cluster the selected GO terms into sub-groups, named super GO terms, which share the similar biological processes (GOs), the GO-GO similarity was calculated using a semantic similarity metric^21^ (implemented using the GOSemSim R package), and then the affinity propagation clustering^22^ (APclustering) model was employed to identify the GO sub-groups, i.e., super-GOs. The number of sub-groups was set 5 empirically.

### Drug repositioning using Connectivity Map (CMAP) and the gene signatures associated with individual super GO terms

The up-regulated genes associated with individual activated GO terms included in each super-GO terms were used as the gene set signatures to identify potentially effective drugs that can inhibit the activated biological function using the connectivity map (CMAP)^15,16^ database. For some super GO terms, there were more than 150 associated up-regulated genes. Since the CMAP suggested the size of gene set signatures should be no more than 150 genes, the genes associated with individual super GO terms were ranked based on the average fold change (of the A549_ACE2 and CALU-3 cells versus the mock condition) in the decreasing manner. Then the top-ranked 150 genes were selected as the gene set signatures to repositioning drugs that can potentially inhibit these up-regulated genes in the gene set signatures. Specifically, the gene set enrichment analysis (GSEA)^23^ was applied on the gene set signatures and the z-profiles (gene expression variation before and after treatment with 2,513 drugs and investigational agents in the CMAP database) of nine cancer cell lines to identify gene set signature-specific inhibitory drugs. Based on the average GSEA scores, the top ranked drugs were selected.

### Up-regulated transcription factors (TFs).

Transcription factors (TFs) are important when exploring regulatory networks. To investigate which set of TFs were up-regulated after SARS-CoV-2 infection in the 2 lung tumor cells use in our study, TF information was derived from the molecular signature database (MSigDB)^24^. Specifically, the C3 category molecular signatures were obtained, which corresponded to regulatory gene sets. From those, 415 TFs interacting with 14802 target genes were identified.

### RdRp-host interaction signaling network inference model.

The protein-protein interaction (PPI) network for these genes was derived from BioGRID^25^ and used as a background signaling network. There were approximately 21,699 genes and 368,918 interactions identified using this approach. The RdRp consequent signaling network inference was defined as a sub-network inference problem within BioGRID. Here, we proposed a novel consequent signaling network inference model. Specifically, let $G_{0}^{i}=\langle R_{i},\emptyset \rangle$ denote the initialized consequent signaling network of root nodes *R*_*i*_. The root nodes are the proteins interacting with the nsp12, nsp7 and nsp8. The growth update of the consequent signaling was defined as: $G_{t+1}^{i}=f\left(G_{t}^{i},G_{B},V_{k},d\right)$, where $G_{t}^{i}$ and *G*_*B*_ is the current down-stream and background (BioGRID) signaling networks, respectively. The edge, *e*_*ij*_ (protein interactions between *g*_*i*_ and *g*_*j*_) of background signaling network, *G*_*B*_, is weighted as: $w\left(e_{ij}\right)=\frac{1}{abs(fc\left(g_{i}\right)}+\frac{1}{abs\left(fc\;(g_{j}\right)}$. *V*_*K*_ and is a vector including K candidate target genes (e.g., the activated genes). For any gene, $g_{k}\in node\left(G_{t}^{i}\right)$, the shortest paths from *g*_*k*_ to the left candidate target genes in *V*_*k*1_, is then calculated. Subsequently, an activation score for each path, *p*_*k,j*_, is defined as: skj=∑gm∈pijfc(gm)n, where *fc(.)* is the fold change calculator and n is the number of genes on the signaling path. If *n* > *d* (search depth parameter) the signaling paths are discarded. In this manner, the parameter k2 decides the search depth. Finally, the signaling path with highest activation score is added to the consequent signaling network. This process is then conducted iteratively until all target nodes are included into the consequent signaling network.

### Identify FDA approved drugs inhibiting genes on the uncovered signaling network.

The FDA approved drug and their target information was derived from drugbank^14^ database. The genes, with degree >= 10, in the RdRp-host signaling network were selected. Then the FDA approved drugs inhibiting the selected genes were identified as drug candidates that can potentially perturb the uncovered signaling network.

## Results

### Activated biological processes in host cells caused by SARS-CoV-2 infection.

As described in the methods section, 656 overlapping (intersection) genes were identified between 1,335 and 2,260 up-regulated genes identified in the CALU-3 and A549_ACE2 cell lines respectively with the fold change >=2.0 and p-value <= 0.05. Via GO enrichment analysis, 474 GO terms in the BP category were identified with a p-value of <= 0.05, and the number of genes in these GO terms was within a range of 10 to 500. Among these identified GO terms, the well-known inflammation and innate immune related GOs terms were identified. As we described in the methods section, given that we aimed to identify the essential host transcription factors that generate proteins which interact with the RdRp of SARS-CoV-2 and its co-factors for viral replications, these inflammation and immune response related GO terms were filtered out empirically. Subsequently, 96 GO terms were finally selected (see supplementary Table 1), in which the 299 activated genes (out of the 656 genes) were included (see supplementary Table 2). As seen, a large set of inflammation and immune response related signaling processes were activated, which indicated the strong inflammation and immune response. In addition, a set of core signaling pathways, like PI3K, ERK1/2, NFkB, JAK-STAT, p38MAPK, JNK, were activated, which are the potential therapeutic targets, like JAK2, for identifying potentially effective COVID-19 treatments. Among the 299 genes, 25 were transcription factors, including: ATF3, ATF4, EGR1, ETS1, FOXO1, FOXO3, GATA3, GATA6, IRF1, IRF2, IRF7, MIER1, NFKBIA, NR1D1, NR4A2, PER1, RORA, RREB1, SKIL, SMAD3, SNAI1, SOX9, STAT1, STAT5A, ZNF175, which indicated the strong effects of SARS-CoV-2 on host regulatory signaling.

**Figure 2:**
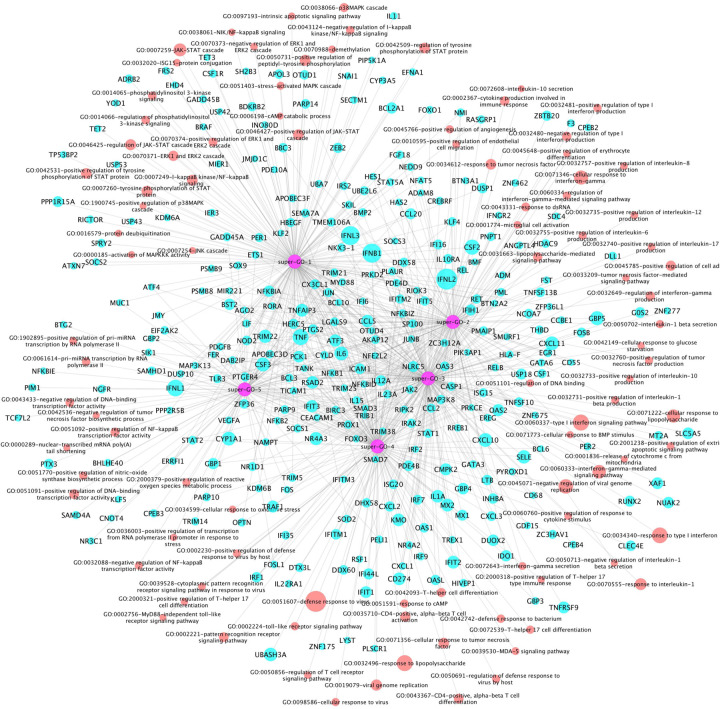
Network of Super GO terms, GO terms, and the associated up-regulated genes, which includes 5 super-GOs (purple), 96 activated GO terms (red) and activated 299 genes (cyan).

Further, the 96 activated GOs were clustered into 5 sub-groups, i.e., super-GO terms (see Fig. 2). There were 25, 20, 21, 19 and 11 GO terms in each of give super GO terms respectively. Accordingly, there were 174, 115, 179, 142 and 86 genes associated with each super GO terms respectively. As seen in Fig. 2, in addition to a set of genes uniquely associated with the 5 super GO terms, some genes were associated with multiple super GO terms. More interestingly, in super GO term-1, there were mainly the core signaling pathways, like JAK-STAT, ERK1/2, NFkB, p38MAPK, PI3K. The interleukin and interferon signaling pathways were included in super GO terms 2 and 3. The T-helper, MyD88, and other immune response signaling pathways were presented in super GO term 4. The DNA-RNA binding, biosynthesis, metabolic signaling pathways were identified in super GO term 5.

### Estrogen hormones inhibiting inflammation and immune response in host cells

**Figure 3:**
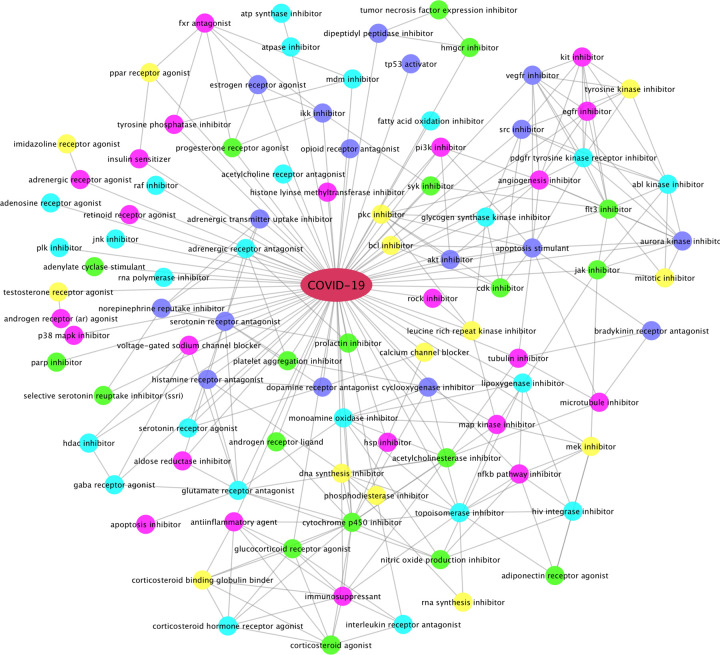
Top-ranked drug categories that can potentially inhibit activated GO terms after SARS-CoV-2 infection. The connection (edge) between two different categories indicates that some drugs can inhibit both categories.

Using the up-regulated genes found in the 5 super-GOs, we identified drugs that can potentially inhibit these activated super-GO terms. Drugs and compounds that had a CMAP drug ranking score of <= −85 (indicating that these drug compounds could inhibit the activated gene set signatures in each super-GO term) were selected. Fig. 3 shows the top-ranked drug categories. Interestingly, the estrogen hormones, estrone (E1) and estriol (E3, weak estrogen hormones), which interact with ESR1/2, as well as estradiol, were top-ranked to be able to inhibit the inflammation and immune response-related biomarker genes in the super-GO terms. As seen, there are a diverse set of drug categories, in addition to *estrogen hormones*, calcium channel blockers, gamma-aminobutyric acid (gaba), androgen, serotonin, adrenergic, glucocorticoid receptor agonist/antagonist; inhibitors of JAK2, MDM2, HDAC, SRC, PI3K, EGFR, RAF, JNK, PLK, p38Mapk, VEGFR, MEK, FLT3, CDK, AKT, HSP, NFkB, IKK; HIV integrase inhibitor, DNA/RNA synthesis inhibitors; and tyrosine, map, aurora kinase inhibitors; corticosteroid agonist, immunosuppressants; SSRI (anti-depression) inhibitors, platelet aggregation inhibitors, as well as tubulin, microtubule inhibitors. Some of these categories have been reported in the new studies about potential COVID-19 treatments. For example, gamma-aminobutyric acid (gaba) administration has been associated with severe illness and death in coronavirus infection in mice^26^. Similarly, calcium channel blockers inhibit SARS-CoV-2 infectivity in lung cells^27^. Moreover, the recent clinical trial results indicated that the baricitinib (the JAK2 inhibitor) plus Remdesivir in combination can significantly reduce the recovery time for and mortality rate of COVID-19 patients receiving high-flow oxygen^28^. These top-ranked drug categories can guide the selection of effective drugs or drug combinations for COVID-19 treatment.

Among the top-ranked drugs based on the super GO term gene set signatures, there were 158 FDA approved drugs. And, 13 of 158 FDA drugs had been evaluated in recent clinical trials: azithromycin, chloroquine, dexamethasone, fluoxetine, formoterol, lenalidomide, losartan, progesterone, ramipril, sildenafil, simvastatin, sitagliptin, tacrolimus, thalidomide and verapamil. Simvastatin^29^, losartan^30^, and chloroquine + azithromycin (used to treat bacterial infections)^31^ have also showed potential efficacy in COVID-19 treatments. SSRI inhibitors and anti-depression drugs, like fluoxetine, were potentially effective for COVID-19 treatment^32^. Ramipril is the ACE inhibitor. The drug dexamethasone, belonging to the glucocorticoid receptor, corticosteroid agonist, and immunosuppressant categories, was the first drug reported to be able to significantly improve the mortality rate of COVID-19 patients in clinical trials^3^, and as such, its inclusion in our results serves as a quasi-experimental positive control. Of note, the National Institutes of Health (NIH) have recently initiated drug combination clinical trials including Remdesivir plus dexamethasone or baricitinib (JAK2 inhibitor) for COVID-19 treatment.

### ESR1 and ESR2 were identified in the viral-host interaction signaling network

**Figure 4:**
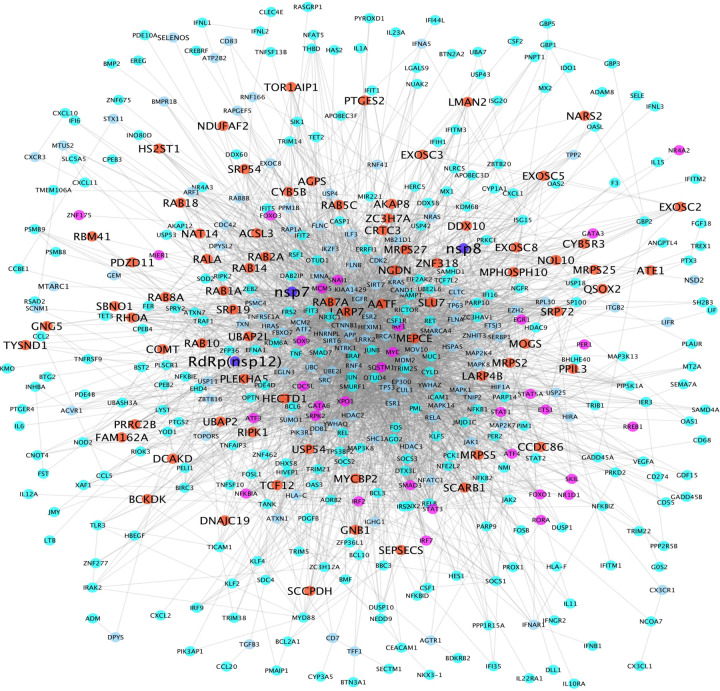
RdRp (nsp12, nsp7 and nsp8)-host interaction signaling network (with 3,373 interactions among 486 proteins). Blue, red, purple, cyan and light blue nodes represent the RdRp-nsp7-nsp8, prey proteins (interacting with RdRp protein complex, transcription factors, up-regulated genes, and proteins linking the prey proteins and up-regulated genes.

Two RdRp-host interaction signaling networks were generated using the proposed network analysis model described in the method section. Supplementary Fig. 1 shows the Nsp12(RdRp)-host interaction signaling network linking the 299 selected genes from 20 prey proteins (root genes) that interact with nsp12 (the RdRp bait protein). There were 22,666 interactions among these 405 genes. Fig. 4 shows the Nsp12-nsp7-nsp8(RdRp)-host interaction signaling network linking the 299 selected genes from 73 prey proteins (root genes) that interact with at least one of nsp12, nsp7, nsp8 (the RdRp bait protein). There were 3,373 interactions among these 486 genes. There are 395 overlapping genes between the two signaling networks, which indicated that the nsp12-host signaling network is a subnetwork of the nsp12-nsp7-nsp8-host signaling network. As seen in the center area of the signaling networks, ESR1 and ESR2, the signaling receptors of estrogen hormones, were identified in the viral-host interaction signaling network, which indicated that ESR1 and ESR2 play important roles in viral-host interactions. In addition, many other signaling targets, e.g., ESR1, ESR2, TP53, HIF1, MYC, HDAC3, TNF, MAPK, RELA, APP, JUN, MDM2, JAK, STAT, CTNNB1, were identified that intensively linked to other signaling targets. These signaling targets linking the prey proteins can be potentially essential viral-host interaction signaling targets, which may work together to facilitate viral replication and regulate host cell signaling response.

### Drugs directly perturb the viral-host interaction signaling networks

**Figure 5:**
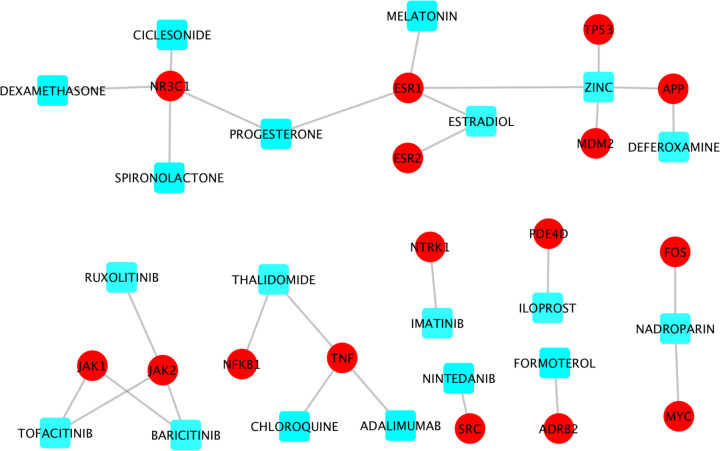
Drug-target information of 19 clinical trials drugs perturbing the RdRp-host interaction signaling network.

There were 200 genes with node degree >=10 in at least one of the two RdRp-host interaction signaling networks (see supplementary Table 3). These genes can be potentially important targets that are synergistic with the RdRp for the purposes of inhibiting viral replication. To identify drugs that can inhibit the targets on this signaling network, drug-target interactions were derived from drugbank^14^ database. Among the 200 genes, there were 65 genes that had at least one drug or investigational agent inhibiting them. Of note, amongst these drugs, 19 were reported in literature concerning clinical trials of potential COVID-19 treatments (i.e., adalimumab, progesterone, spironolactone, chloroquine, imatinib, deferoxamine, estradiol, formoterol, thalidomide, melatonin, iloprost, dexamethasone, ciclesonide, zinc, nadroparin, ruxolitinib, tofacitinib, nintedanib, and baricitinib) (see Fig. 5). Interestingly, nadroparin, the MYC and FOS inhibitor, can stop the amplification of the fibrin clotting cascade. Similarly, thalidomide is a NFkB and TNF inhibitor that can be immune-suppressive and anti-angiogenic^33^. Estradiol and melatonin^34^ are ESR1 inhibitors that tested in clinical trials. Also, dexamethasone, NR3C1 inhibitor, was the first drug reported to be able to significantly improve the mortality rate of COVID-19 patients in clinical trials^3^. The newly clinical trial results showed that Baricitnib, the JAK2 inhibitor, was synergistic with Remdesivir to improve the outcome of COVID-19 patients^28^.

Moreover, the selected drugs (some targets have many drugs directly interacting with them) that can perturb one of the 65 signaling targets were shown in Supplementary Fig. 2. Some targets have been reported in the current literature as being potentially important and effective targets for COVID-19 treatment (in addition to drugs that could modulate the effects of cytokines), like HDAC2/3^35,36^, JAK2^37^, CDK2^38^, AKT1^39^, MDM2^40^, TNF^41^, MAPK14^9^, STAT3^42^, BCL2^43^, NFKB1A^44^, EGFR^42^. Inhibitors (drugs) of these targets can be potentially synergistic with Remdesivir to inhibit viral replication.

### Novel evidence showing that estrogen hormone is an essential sex factor causing the distinct fatality patterns between male and female COVID-19 patients

**Figure 6:**
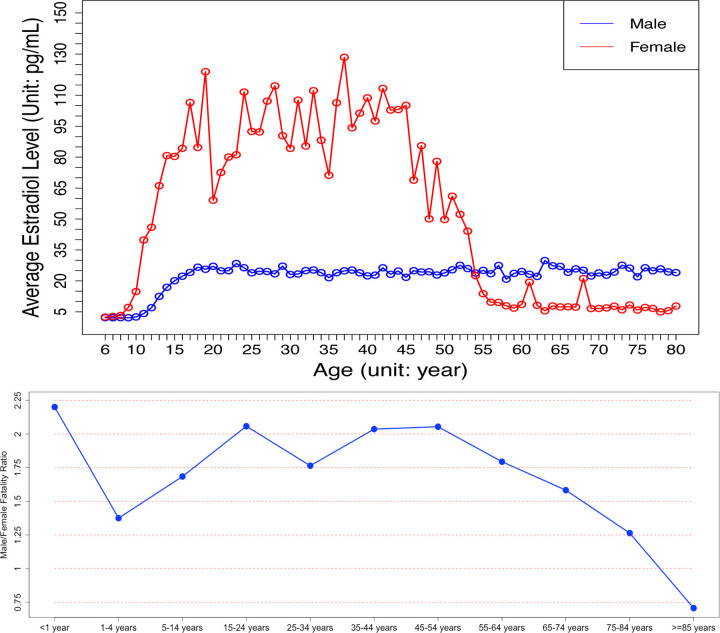
The estrogen level lifespan changes in women and men (upper-panel), and the curve of ratio between female and male mortality rate of COVID-19 patients (bottom-panel).

As shown in the results, the estrogen hormones can inhibit the activated inflammation and immune response biomarker genes; and the ESR1 and ESR2, the signaling receptors of estrogen hormones, were identified in the viral-host signaling network and intensively interact with other signaling targets on the network. All the results indicated that the estrogen hormones, interacting with ESR1/ESR2, are the essential sex factors protecting young female COVID-19 patients. However, the fatality rate of old female COVID-19 patients dramatically increased. To further understand the mechanism of estrogen hormones, we investigated the lifespan changes of the estrogen level in women and men^45^. Very importantly, it showed that the estrogen level in female reduces to an extremely low level after about 55 years of age, starting from about 45 years of age (see Fig. 6–**upper panel**). Of note, we further downloaded the mortality data of male and female (see Fig. 6–**bottom panel**) in U.S. from the CDC website^46^ (by December 31, 2020), and compared those data with the estrogen levels of female over their life span^45^ (see Fig. 6–**upper panel**). Surprisingly, the trends of the two curves closely approximated each-other (i.e., the curve of ratio between female and male mortality rate of COVID-19 patients vs the cure of levels of female). This specific findings might be able to explain, in terms of the molecular mechanism, the mounting evidence suggesting that men have a significantly higher mortality rate when positive for COVID-19 than young women^47^. In other words, the low level of estoren hormone in female before 9 years old, and the dramatic decreasing of estradiol after ~55 years of age is associated with an increasing COVID-19 mortality rate in female. This may serve an indicator that estrogens can serves as an effective treatment, especially for old male or female patients, or in combination with Remdesivir for COVID-19 treatment.

## 4. Discussion

Millions of people are being infected by SARS-CoV-2 globally, with thousands of ensuing deaths every day. There is currently a lack of effective treatments to reduce the mortality rate of COVID-19. Further, given the slow rates of vaccine delivery throughout the glove, and the emergence of new SARS-CoV-2 variants, the need for new and effective treatments is both critical and unlikely to cease to be a priority for the foreseeable future. Remdesivir is a widely used and promising drug inhibiting the RdRp protein. However, the evidence of efficacy of using Remdesivir alone is limited. To improve the efficacy of Remdesivir, one possible approach is to combine Remdesivir with other drugs that can inhibit the down-stream signaling of RdRp.

Sex had been indicated as an essential factor in the mortality of COVID-19. The global data of COVID-19 showed that men have a higher mortality rate than women. Therefore, the sex difference indicated that sex related genetic factors could be an interesting mechanism and targets for COVID-19 treatment, combined with existing treatment options. However, the underlying targets and signaling pathways associated with the sex difference remain unclear. Using the systematic and integrative network analysis models, we identified estrogen hormones (ER), interacting with ESR1 and ESR2, are essential sex factors causing distinct mortality patterns in female and male COVID-19 patients. Specifically, a high level of estradiol protects young female COVID-19 patients, and estrogen loss to an extremely low level in females after about 55 years of age causes the increased fatality rate of women. Moreover, estrogen hormones can be effective treatments for COVID-19 by inhibiting the inflammation and immune response within host cells caused by SARS-CoV-2. These novel molecular mechanisms could provide clues to develop novel and effective treatments for COVID-19. One challenge is that it remains unclear and difficult to identify the ‘causal’ down-stream signaling interactions of the sex associated genetic factors among the host signaling network to regulate inflammation and immune response. For example, it is interesting and important to further study the molecular mechanism of how the ESR1 and ESR2, or the other essential sex associated genetic factors interact with other signaling targets to regulate host cell response (like inflammation and immune response).

## Conclusion

In this study, we conclude that estrogen hormone is an essential sex factor causing the distinct fatality rates between male and female COVID-19 patients. Specifically, a high level of estradiol protecting young female COVID-19 patients, and estrogen loss to an extremely low level in females after about 55 years of age causing the increased fatality rate of women. In addition, the uncovered essential host targets and treatments can help designing experimental validations to uncover effective targets and drugs along or combined with Remdesivir as novel treatment regimens of COVID-19.

## Supplementary Material

Supplement 1

Supplement 2
