# Supplementary material for: Estrogen hormone is an essential sex factor inhibiting inflammation and immune response in COVID-19
Source: Res Sq. 2021 Sep 30:rs.3.rs-936900. Preprint. [Version 1] doi: 10.21203/rs.3.rs-936900/v1 (PMC8491851; doi:10.21203/rs.3.rs-936900/v1)
Supplement: Supplement 1 [file 6186e9d8e594ed77c4f0a8b1.docx]

**Supplementary Figures**


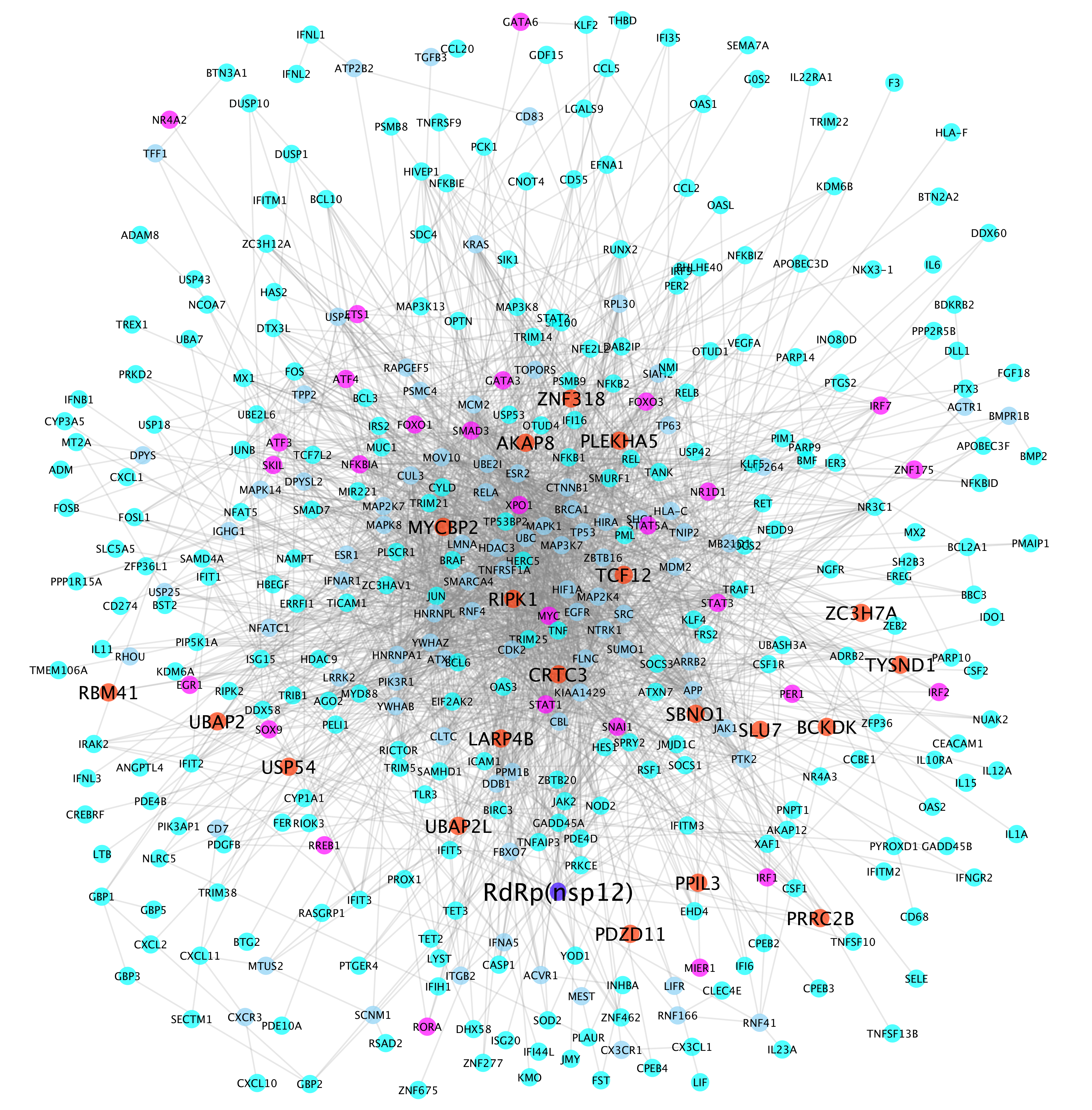


**Supplementary Figure 1**: Nsp12-host interaction signaling network (with 2,266 protein-protein interactions among 405 proteins). Blue, red, purple, cyan and light blue nodes represent the RdRp(nsp12), prey proteins interacting with nsp12, transcription factors, up-regulated genes, and proteins linking the prey proteins and up-regulated genes.


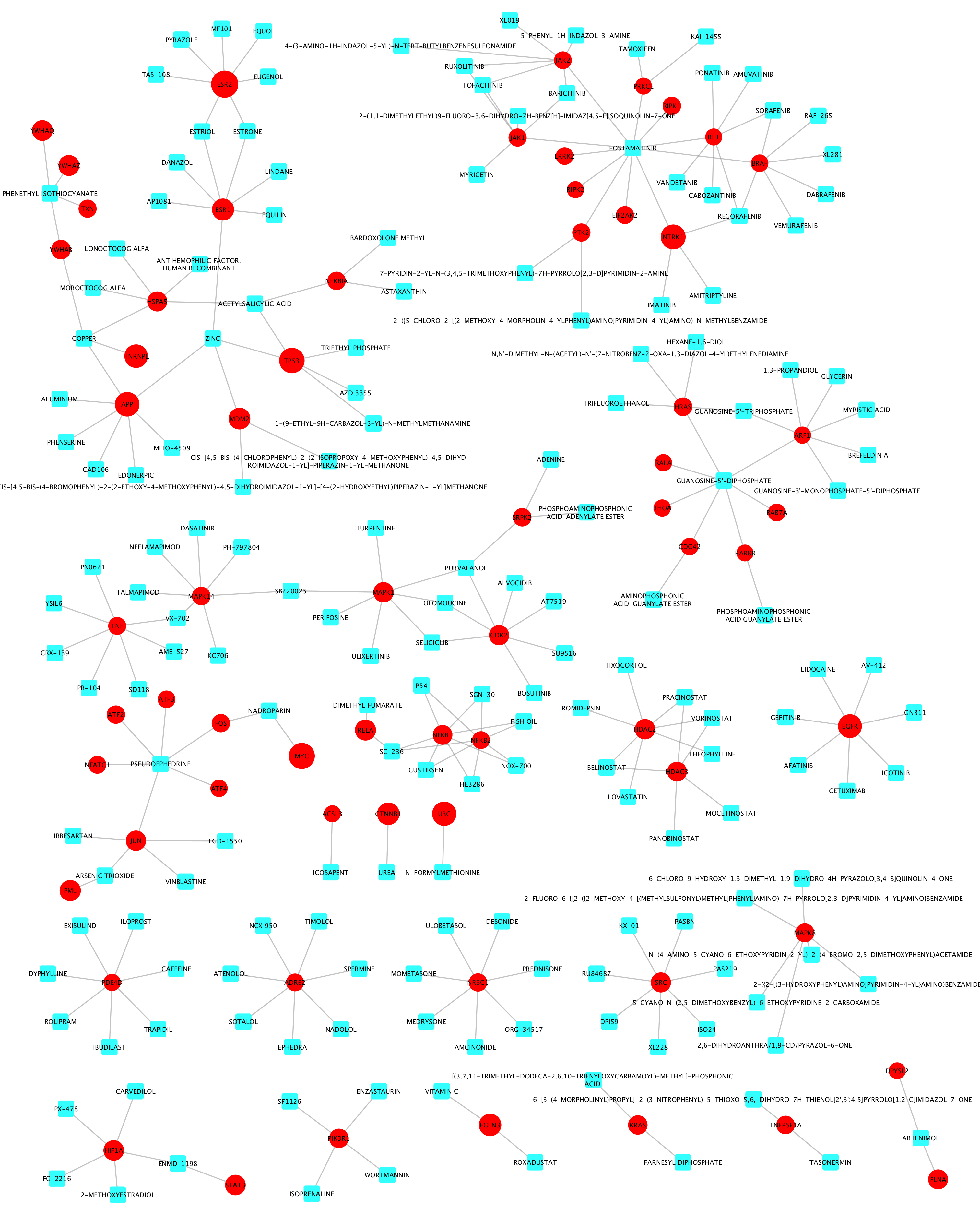


**Supplementary Figure 2**: Selected drugs perturbing the 65 signaling targets on the RdRP-host interaction signaling networks. Red and Cyan nodes represent targets and drugs respectively.
