# Supplementary material for: Estrogen hormone is an essential sex factor inhibiting inflammation and immune response in COVID-19
Source: Res Sq. 2021 Sep 30:rs.3.rs-936900. Preprint. [Version 1] doi: 10.21203/rs.3.rs-936900/v1 (PMC8491851; doi:10.21203/rs.3.rs-936900/v1)
Supplement: Supplement 2 [file 455069091d37f8684306fba7.docx]

**Supplementary Table 1:** Activated biological process (BP) gene ontology (GO) terms in both lung tumor cells after SARS-CoV-2 infection.

| **Go_id** | **Go_term_name** | **# of genes** | **P_value** |
| --- | --- | --- | --- |
| GO:0032020 | ISG15-protein conjugation | 11 | 0.00012678 |
| GO:0032480 | negative regulation of type I interferon production | 49 | 2.64E-09 |
| GO:0032649 | regulation of interferon-gamma production | 120 | 1.90E-09 |
| GO:0034340 | response to type I interferon | 125 | 1.60E-25 |
| GO:0042742 | defense response to bacterium | 392 | 7.38E-07 |
| GO:0045071 | negative regulation of viral genome replication | 70 | 2.25E-22 |
| GO:0045648 | positive regulation of erythrocyte differentiation | 32 | 0.00159033 |
| GO:0051607 | defense response to virus | 302 | 8.30E-39 |
| GO:0060337 | type I interferon signaling pathway | 116 | 2.63E-26 |
| GO:0072608 | interleukin-10 secretion | 13 | 0.00497287 |
| GO:0072643 | interferon-gamma secretion | 23 | 3.42E-05 |
| GO:0033209 | tumor necrosis factor-mediated signaling pathway | 197 | 0.00141183 |
| GO:0042536 | negative regulation of tumor necrosis factor biosynthetic process | 10 | 0.00304971 |
| GO:0070373 | negative regulation of ERK1 and ERK2 cascade | 79 | 0.00014611 |
| GO:0001836 | release of cytochrome c from mitochondria | 75 | 0.00386129 |
| GO:0097193 | intrinsic apoptotic signaling pathway | 376 | 0.00160055 |
| GO:0098586 | cellular response to virus | 61 | 4.12E-06 |
| GO:0002230 | positive regulation of defense response to virus by host | 35 | 1.12E-05 |
| GO:0010595 | positive regulation of endothelial cell migration | 151 | 5.24E-05 |
| GO:0016579 | protein deubiquitination | 443 | 0.00215326 |
| GO:0032088 | negative regulation of NF-kappaB transcription factor activity | 98 | 4.30E-06 |
| GO:0034599 | cellular response to oxidative stress | 365 | 0.00037985 |
| GO:0042149 | cellular response to glucose starvation | 46 | 0.00111448 |
| GO:0043124 | negative regulation of I-kappaB kinase/NF-kappaB signaling | 58 | 1.66E-06 |
| GO:0045766 | positive regulation of angiogenesis | 242 | 3.52E-06 |
| GO:0050713 | negative regulation of interleukin-1 beta secretion | 15 | 0.00154482 |
| GO:0071222 | cellular response to lipopolysaccharide | 231 | 4.82E-19 |
| GO:0071356 | cellular response to tumor necrosis factor | 333 | 1.44E-12 |
| GO:1900745 | positive regulation of p38MAPK cascade | 35 | 0.00393027 |
| GO:2000379 | positive regulation of reactive oxygen species metabolic process | 112 | 2.74E-06 |
| GO:0001774 | microglial cell activation | 55 | 5.35E-05 |
| GO:0032496 | response to lipopolysaccharide | 366 | 4.65E-26 |
| GO:0051591 | response to cAMP | 106 | 1.81E-06 |
| GO:0070374 | positive regulation of ERK1 and ERK2 cascade | 254 | 5.32E-08 |
| GO:1902895 | positive regulation of pri-miRNA transcription by RNA polymerase II | 35 | 3.89E-05 |
| GO:0006198 | cAMP catabolic process | 10 | 0.00497287 |
| GO:0000185 | activation of MAPKKK activity | 14 | 0.00148463 |
| GO:0002224 | toll-like receptor signaling pathway | 201 | 1.91E-06 |
| GO:0007249 | I-kappaB kinase/NF-kappaB signaling | 361 | 1.03E-12 |
| GO:0031663 | lipopolysaccharide-mediated signaling pathway | 61 | 4.03E-09 |
| GO:0050856 | regulation of T cell receptor signaling pathway | 44 | 0.00030531 |
| GO:0051092 | positive regulation of NF-kappaB transcription factor activity | 193 | 1.54E-08 |
| GO:2001238 | positive regulation of extrinsic apoptotic signaling pathway | 57 | 7.28E-05 |
| GO:0071346 | cellular response to interferon-gamma | 202 | 2.06E-21 |
| GO:0060333 | interferon-gamma-mediated signaling pathway | 99 | 7.02E-20 |
| GO:0050702 | interleukin-1 beta secretion | 67 | 2.81E-06 |
| GO:0014066 | regulation of phosphatidylinositol 3-kinase signaling | 138 | 4.03E-07 |
| GO:0050731 | positive regulation of peptidyl-tyrosine phosphorylation | 225 | 3.98E-13 |
| GO:0036003 | positive regulation of transcription from RNA polymerase II promoter in response to stress | 47 | 0.00096897 |
| GO:0032481 | positive regulation of type I interferon production | 108 | 1.82E-06 |
| GO:0032731 | positive regulation of interleukin-1 beta production | 63 | 1.95E-05 |
| GO:0034612 | response to tumor necrosis factor | 359 | 1.03E-12 |
| GO:0070555 | response to interleukin-1 | 233 | 2.30E-15 |
| GO:0043433 | negative regulation of DNA-binding transcription factor activity | 193 | 1.91E-07 |
| GO:0014065 | phosphatidylinositol 3-kinase signaling | 176 | 3.49E-07 |
| GO:0071773 | cellular response to BMP stimulus | 256 | 0.00082907 |
| GO:0051403 | stress-activated MAPK cascade | 372 | 8.97E-06 |
| GO:0038061 | NIK/NF-kappaB signaling | 202 | 0.00086822 |
| GO:0045785 | positive regulation of cell adhesion | 489 | 2.33E-11 |
| GO:0039530 | MDA-5 signaling pathway | 11 | 2.47E-05 |
| GO:0051091 | positive regulation of DNA-binding transcription factor activity | 322 | 1.69E-10 |
| GO:0002221 | pattern recognition receptor signaling pathway | 262 | 3.63E-10 |
| GO:0061614 | pri-miRNA transcription by RNA polymerase II | 52 | 7.22E-07 |
| GO:0002756 | MyD88-independent toll-like receptor signaling pathway | 38 | 0.00368722 |
| GO:0046427 | positive regulation of JAK-STAT cascade | 106 | 1.86E-12 |
| GO:0032733 | positive regulation of interleukin-10 production | 40 | 0.00133082 |
| GO:0032735 | positive regulation of interleukin-12 production | 36 | 8.96E-05 |
| GO:0032740 | positive regulation of interleukin-17 production | 17 | 5.31E-05 |
| GO:0032760 | positive regulation of tumor necrosis factor production | 95 | 6.41E-06 |
| GO:0042509 | regulation of tyrosine phosphorylation of STAT protein | 94 | 1.86E-12 |
| GO:0042531 | positive regulation of tyrosine phosphorylation of STAT protein | 80 | 1.66E-12 |
| GO:2000318 | positive regulation of T-helper 17 type immune response | 12 | 0.00154482 |
| GO:0007259 | JAK-STAT cascade | 209 | 2.01E-18 |
| GO:0000289 | nuclear-transcribed mRNA poly(A) tail shortening | 50 | 0.00303652 |
| GO:0038066 | p38MAPK cascade | 59 | 1.90E-08 |
| GO:0070371 | ERK1 and ERK2 cascade | 375 | 1.24E-11 |
| GO:0072539 | T-helper 17 cell differentiation | 27 | 1.95E-06 |
| GO:0070988 | demethylation | 108 | 6.97E-05 |
| GO:0060334 | regulation of interferon-gamma-mediated signaling pathway | 27 | 5.80E-06 |
| GO:0002367 | cytokine production involved in immune response | 111 | 1.40E-09 |
| GO:0007254 | JNK cascade | 287 | 0.00495514 |
| GO:0032755 | positive regulation of interleukin-6 production | 107 | 5.03E-06 |
| GO:0032757 | positive regulation of interleukin-8 production | 57 | 0.00016468 |
| GO:0051770 | positive regulation of nitric-oxide synthase biosynthetic process | 16 | 4.83E-06 |
| GO:0019079 | viral genome replication | 138 | 5.52E-14 |
| GO:0043331 | response to dsRNA | 60 | 5.96E-08 |
| GO:2000321 | positive regulation of T-helper 17 cell differentiation | 10 | 0.00064445 |
| GO:0051101 | regulation of DNA binding | 136 | 0.00011142 |
| GO:0035710 | CD4-positive, alpha-beta T cell activation | 105 | 2.92E-11 |
| GO:0043367 | CD4-positive, alpha-beta T cell differentiation | 85 | 4.12E-10 |
| GO:0039528 | cytoplasmic pattern recognition receptor signaling pathway in response to virus | 32 | 6.27E-05 |
| GO:0060760 | positive regulation of response to cytokine stimulus | 61 | 9.02E-05 |
| GO:0007260 | tyrosine phosphorylation of STAT protein | 104 | 2.26E-13 |
| GO:0046425 | regulation of JAK-STAT cascade | 165 | 2.98E-13 |
| GO:0050691 | regulation of defense response to virus by host | 46 | 4.18E-06 |
| GO:0042093 | T-helper cell differentiation | 66 | 1.90E-08 |

**Supplementary Table 2:** The 299 up-regulated genes, in both CA549_ACE2 and CALU-3 cell lines, associated with the 96 selected activated GO terms.

| ADAM8 | CNOT4 | FOSB | IFITM3 | LYST | PARP10 | RREB1 | TNFRSF9 |
| --- | --- | --- | --- | --- | --- | --- | --- |
| ADM | CPEB2 | FOSL1 | IFNB1 | MAP3K13 | PARP14 | RSAD2 | TNFSF10 |
| ADRB2 | CPEB3 | FOXO1 | IFNGR2 | MAP3K8 | PARP9 | RSF1 | TNFSF13B |
| AGO2 | CPEB4 | FOXO3 | IFNL1 | MIER1 | PCK1 | RUNX2 | TP53BP2 |
| AKAP12 | CREBRF | FRS2 | IFNL2 | MIR221 | PDE10A | SAMD4A | TRAF1 |
| ANGPTL4 | CSF1 | FST | IFNL3 | MT2A | PDE4B | SAMHD1 | TREX1 |
| APOBEC3D | CSF1R | G0S2 | IL10RA | MUC1 | PDE4D | SDC4 | TRIB1 |
| APOBEC3F | CSF2 | GADD45A | IL11 | MX1 | PDGFB | SECTM1 | TRIM14 |
| APOL3 | CSF3 | GADD45B | IL12A | MX2 | PELI1 | SELE | TRIM21 |
| ATF3 | CX3CL1 | GATA3 | IL15 | MYD88 | PER1 | SEMA7A | TRIM22 |
| ATF4 | CXCL1 | GATA6 | IL1A | NAMPT | PER2 | SH2B3 | TRIM25 |
| ATXN7 | CXCL10 | GBP1 | IL22RA1 | NCOA7 | PIK3AP1 | SIK1 | TRIM38 |
| BBC3 | CXCL11 | GBP2 | IL23A | NEDD9 | PIM1 | SKIL | TRIM5 |
| BCL10 | CXCL2 | GBP3 | IL6 | NFAT5 | PIP5K1A | SLC5A5 | UBA7 |
| BCL2A1 | CXCL3 | GBP4 | INHBA | NFE2L2 | PLAUR | SMAD3 | UBASH3A |
| BCL3 | CYLD | GBP5 | INO80D | NFKB1 | PLSCR1 | SMAD7 | UBE2L6 |
| BCL6 | CYP1A1 | GDF15 | IRAK2 | NFKB2 | PMAIP1 | SMURF1 | USP18 |
| BDKRB2 | CYP3A5 | HAS2 | IRF1 | NFKBIA | PML | SNAI1 | USP42 |
| BHLHE40 | DAB2IP | HBEGF | IRF2 | NFKBID | PNPT1 | SOCS1 | USP43 |
| BIRC3 | DDX58 | HDAC9 | IRF7 | NFKBIE | PPP1R15A | SOCS2 | USP53 |
| BMF | DDX60 | HERC5 | IRF9 | NFKBIZ | PPP2R5B | SOCS3 | VEGFA |
| BMP2 | DHX58 | HES1 | IRS2 | NGFR | PRKCE | SOD2 | XAF1 |
| BRAF | DLL1 | HIVEP1 | ISG15 | NKX3-1 | PRKD2 | SOX9 | YOD1 |
| BST2 | DTX3L | HLA-F | ISG20 | NLRC5 | PROX1 | SP100 | ZBTB20 |
| BTG2 | DUOX2 | ICAM1 | JAK2 | NMI | PSMB8 | SPRY2 | ZC3H12A |
| BTN2A2 | DUSP1 | IDO1 | JMJD1C | NOD2 | PSMB9 | STAT1 | ZC3HAV1 |
| BTN3A1 | DUSP10 | IER3 | JMY | NR1D1 | PTGER4 | STAT2 | ZEB2 |
| CASP1 | EFNA1 | IFI16 | JUN | NR3C1 | PTGS2 | STAT5A | ZFP36 |
| CCBE1 | EGR1 | IFI35 | JUNB | NR4A2 | PTX3 | TANK | ZFP36L1 |
| CCL2 | EHD4 | IFI44L | KDM6A | NR4A3 | PYROXD1 | TCF7L2 | ZNF175 |
| CCL20 | EIF2AK2 | IFI6 | KDM6B | NUAK2 | RASGRP1 | TET2 | ZNF277 |
| CCL5 | EREG | IFIH1 | KLF2 | OAS1 | REL | TET3 | ZNF462 |
| CD274 | ERRFI1 | IFIT1 | KLF4 | OAS2 | RELB | THBD | ZNF675 |
| CD55 | ETS1 | IFIT2 | KLF5 | OAS3 | RET | TICAM1 |  |
| CD68 | F3 | IFIT3 | KMO | OASL | RICTOR | TLR3 |  |
| CEACAM1 | FER | IFIT5 | LGALS9 | OPTN | RIOK3 | TMEM106A |  |
| CLEC4E | FGF18 | IFITM1 | LIF | OTUD1 | RIPK2 | TNF |  |
| CMPK2 | FOS | IFITM2 | LTB | OTUD4 | RORA | TNFAIP3 |  |

**Supplementary Table 3**: The 200 genes with node degree >=10 in at least one of the RdRp-host interaction signaling networks. The number in the parentheses indicates the maximum degree of individual genes in the two signaling networks.

| KIAA1429(149) | LARP7(42) | ZC3HAV1(25) | MAP2K7(16) | RET(12) |
| --- | --- | --- | --- | --- |
| ESR2(109) | SRC(41) | ATF2(25) | FOXO3(16) | IFIT3(12) |
| TRIM25(99) | SMARCA4(41) | SHC1(24) | UBAP2L(16) | STAT2(12) |
| MYC(98) | LMNA(41) | NR3C1(24) | MAP2K4(15) | ETS1(12) |
| TP53(92) | HSPA5(41) | PSMC4(24) | SOCS1(15) | PLSCR1(12) |
| NTRK1(87) | FLNA(40) | PTK2(23) | SMAD7(15) | HIVEP1(12) |
| EP300(85) | HNRNPA1(39) | JAK1(23) | RUNX2(15) | NRAS(12) |
| APP(84) | STAT1(39) | RICTOR(23) | USP4(15) | IKZF3(12) |
| XPO1(83) | HDAC3(38) | TXN(23) | ATF4(15) | SIRT6(12) |
| UBC(81) | NFKB1(38) | USP11(23) | KLF5(15) | OPTN(12) |
| EGFR(77) | CYLD(38) | ARRB2(22) | MYCBP2(15) | RAB10(12) |
| HNRNPL(74) | MAP3K7(37) | FLNC(22) | RAP1A(15) | ACSL3(12) |
| BRCA1(68) | DDB1(37) | BIRC3(21) | PDE4D(14) | PPM1B(11) |
| RNF4(65) | YWHAB(36) | ZBTB16(21) | HIRA(14) | DDX58(11) |
| EGLN3(65) | PIK3R1(36) | TRAF1(21) | TANK(14) | NMI(11) |
| CTNNB1(64) | IFI16(36) | EIF2AK2(21) | BCL3(14) | PRKCE(11) |
| ESR1(60) | KRAS(35) | FTSJ3(21) | NFE2L2(14) | IRF7(11) |
| MDM2(57) | MB21D1(34) | RAB1A(21) | IRS2(14) | OTUD4(11) |
| CUL3(55) | MEPCE(34) | TNF(20) | RELB(14) | RAB8A(11) |
| CAND1(53) | CBL(32) | TRIM21(20) | HLA-C(14) | RAB14(11) |
| PML(52) | SUMO1(32) | ADRB2(20) | ARF1(14) | FOXO1(11) |
| MCM2(52) | CLTC(32) | NFATC1(20) | RHOA(14) | PIP5K1A(11) |
| UBE2I(51) | ISG15(31) | JAK2(19) | RALA(14) | DPYSL2(11) |
| MOV10(51) | CDC5L(31) | NFKB2(19) | EXOSC8(14) | RAB8B(11) |
| RELA(50) | SMURF1(30) | AGO2(19) | RAB2A(14) | OTUD1(11) |
| SQSTM1(50) | MAPK8(30) | JUNB(19) | MRPS5(14) | ZNF318(11) |
| YWHAZ(49) | FBXO7(28) | TP63(19) | GNB1(14) | MRPS2(11) |
| YWHAQ(48) | REL(28) | RAB5C(19) | HECTD1(14) | TOPORS(10) |
| HEXIM1(47) | SNAI1(28) | FLNB(19) | ATXN1(13) | SPRY2(10) |
| STAT3(46) | MCM5(28) | MRPS27(19) | SIAH2(13) | MX1(10) |
| SMAD3(46) | HRAS(28) | ATF3(18) | MYD88(13) | TCF12(10) |
| HIF1A(46) | SERBP1(28) | LRRK2(18) | UBE2L6(13) | EXOSC5(10) |
| TNIP2(46) | TNFRSF1A(27) | RAB7A(18) | AKAP8(13) | ZFP36(10) |
| MAPK1(44) | NFKBIA(26) | SRP72(18) | DAB2IP(13) | MRPS25(10) |
| JUN(44) | MAPK14(26) | STAT5A(17) | PLEKHA5(13) | MOGS(10) |
| HDAC2(44) | SRPK2(26) | SAMHD1(17) | TRIM14(13) | AKAP12(10) |
| EZH2(43) | RIPK1(25) | BCL6(17) | AATF(13) | HERC5(10) |
| CDK2(42) | TNFAIP3(25) | BRAF(17) | DDX10(13) | TCF7L2(10) |
| ILF3(42) | FOS(25) | CDC42(17) | TP53BP2(12) | EXOSC3(10) |
| SIRT7(42) | RPL30(25) | SOCS3(16) | RIPK2(12) | SRP54(10) |
